# Analysis of the efficacy of Taiwanese freeze-dried neurotoxic antivenom against *Naja kaouthia*, *Naja siamensis* and *Ophiophagus hannah* through proteomics and animal model approaches

**DOI:** 10.1371/journal.pntd.0006138

**Published:** 2017-12-15

**Authors:** Chien-Chun Liu, Chen-Hsien You, Po-Jung Wang, Jau-Song Yu, Guo-Jen Huang, Chien-Hsin Liu, Wen-Chin Hsieh, Chih-Chuan Lin

**Affiliations:** 1 Graduate Institute of Biomedical Sciences, College of Medicine, Chang Gung University, Tao-Yuan, Taiwan; 2 Department of Medicine, College of Medicine, Chang Gung University, Tao-Yuan, Taiwan; 3 Department of Cell and Molecular Biology, College of Medicine, Chang Gung University, Tao-Yuan, Taiwan; 4 Molecular Medicine Research Center, Chang Gung University, Tao-Yuan, Taiwan; 5 Liver Research Center, Chang Gung Memorial Hospital, Linkou, Tao-Yuan, Taiwan; 6 Center for Research, Diagnostics and Vaccine Development, Centers for Disease Control, Ministry of Health and Welfare, Taipei, Taiwan; 7 Department of Emergency Medicine, Chang Gung Memorial Hospital, Linkou, Tao-Yuan, Taiwan; University of Newcastle, AUSTRALIA

## Abstract

In Southeast Asia, envenoming resulting from cobra snakebites is an important public health issue in many regions, and antivenom therapy is the standard treatment for the snakebite. Because these cobras share a close evolutionary history, the amino acid sequences of major venom components in different snakes are very similar. Therefore, either monovalent or polyvalent antivenoms may offer paraspecific protection against envenomation of humans by several different snakes. In Taiwan, a bivalent antivenom—freeze-dried neurotoxic antivenom (FNAV)—against *Bungarus multicinctus* and *Naja atra* is available. However, whether this antivenom is also capable of neutralizing the venom of other species of snakes is not known. Here, to expand the clinical application of Taiwanese FNAV, we used an animal model to evaluate the neutralizing ability of FNAV against the venoms of three common snakes in Southeast Asia, including two ‘true’ cobras *Naja kaouthia* (Thailand) and *Naja siamensis* (Thailand), and the king cobra *Ophiophagus hannah* (Indonesia). We further applied mass spectrometry (MS)-based proteomic techniques to characterize venom proteomes and identify FNAV-recognizable antigens in the venoms of these Asian snakes. Neutralization assays in a mouse model showed that FNAV effectively neutralized the lethality of *N*. *kaouthia* and *N*. *siamensis* venoms, but not *O*. *hannah* venom. MS-based venom protein identification results further revealed that FNAV strongly recognized three-finger toxin and phospholipase A2, the major protein components of *N*. *kaouthia* and *N*. *siamensis* venoms. The characterization of venom proteomes and identification of FNAV-recognizable venom antigens may help researchers to further develop more effective antivenom designed to block the toxicity of dominant toxic proteins, with the ultimate goal of achieving broadly therapeutic effects against these cobra snakebites.

## Author summary

Cobra envenomation is a public health issue in Southeast Asia. Currently, antivenom therapy is the standard treatment for snakebite. However, antivenoms are not available in many rural countries and communities or have only limited effectiveness. Taiwan has wealth of experience in producing antivenoms, including the bivalent freeze-dried neurotoxic antivenom (FNAV), which is raised against venom proteins from *Bungarus multicinctus* and *Naja atra*. Our results showed that FNAV effectively neutralized the lethality of *Naja kaouthia*(Thailand) and *Naja siamensis* (Thailand) venoms, but not *Ophiophagus hannah* (Indonesia) venom, in an animal model. We further characterized the venom proteome profiles of the four cobras and identified three abundant proteins—neurotoxin, cytotoxin and phospholipase A2—in the venom of *N*. *atra*, *N*. *kaouthia* and *N*. *siamensis*as the major antigens recognized by FNAV. In contrast, we found that β-cardiotoxin and phospholipase A2, common toxin proteins in all king cobra venom samples, are weakly or not recognized by FNAV. Our data provide evidence suggesting the potential use of Taiwan’s FNAV to treat envenomation by other cobra species (*N*. *kaouthia* and *N*. *siamensis*) in Southeast Asia. Moreover, our findings support the previous recommendation and current experimental approach that major cobra toxins are used as antigens to generate more efficient antivenoms than those currently available.

## Introduction

Envenomation through snakebite is an important public health issue in many regions of the world, particularly in tropical countries [1–3]. An estimated 421,000 to 1,841,000 envenomations and 20,000 to 94,000 deaths occur globally each year owing to snakebites. The regions of highest incidence include Southeast Asia, South Asia, Africa, and Latin America [4]. In Southeast Asia, cases involving cobra envenomation are among the most common[5]. There are several clinically significant cobra snakes: *Naja atra*, *Naja kaouthia*, *Naja siamensis*, *Naja sputatrix*, *Naja sumatrana*, and *Naja philipinensis*.

At present, antivenom therapy is the standard treatment for snakebite. To maximize antivenom utility, researchers have applied animal models to evaluate the ability of antivenoms to cross-neutralize the venoms of other snakes in the same genus that represent a public health concern [6, 7]. These approaches, combined with immunological and proteomics techniques, have been successfully used to identify specific venom proteins that can be recognized by antivenom [8–11]. Such information can be used to design a new strategy for improving the immune response of animals against poorly immunogenic antigens or major toxic components so as to further improve the efficacy of antivenoms [12–14].

There are four types of available antivenom against the six most clinically significant snakebites in Taiwan; two are bivalent antivenoms, and the other two are monovalent antivenoms [15, 16]. One of the bivalent antivenoms is freeze-dried neurotoxic antivenom (FNAV), raised against *Bungarus multicinctus* and *N*. *atra*. Previous studies have shown that FNAV exhibits good clinical effects and is well documented to decrease the rate of death caused by bites from these two snakes [17, 18].

The aim of this study was to evaluate whether FNAV has therapeutic potential for envenomations of cobra species outside of Taiwan. In this preclinical study, we analyzed the ability of FNAV to neutralize the venoms of *N*. *kaouthia*, *N*. *siamensis* and *O*. *hannah*. We further investigated the venom proteome of each cobra by liquid chromatography-tandem mass spectrometry (LC-MS/MS) analysis and identified FNAV-recognizable components in each. These results not only provide useful information regarding the neutralizing potential of FNAV against heterologous venoms, it also provides valuable clues for improving antivenom efficacy.

## Methods

### Snake venoms and antivenoms

The lyophilized venom of *N*. *atra* was obtained from the Centers for Disease Control, R.O.C (Taiwan). Venoms of two other Southeast Asia *Naja* species, *N*. *kaouthia* and *N*. *siamensis*, as well as that of the related king cobra, *O*. *hannah* (the sole member of its genus), were purchased from Latoxan (Valence, France). According to Latoxan’s remark, the snakes of *N*. *kaouthia*, *N*. *siamensis* and *O*. *hannah* originate from Thailand, Thailand and Indonesia, respectively. Venoms were collected from several adult specimens, then freeze-dried and stored at -20°C before use. Freeze neurotoxic (FN) antivenoms were purchased as lyophilized powders from the Centers for Disease Control, R.O.C (Taiwan), and stored at 4°C before use.

### Animals

Experiments were performed on 7-week-old littermate male mice (C57BL/6Narl strain, 20–25 g). Mice were maintained under specific pathogen-free conditions with a 12:12 hour light-dark cycle at a temperature of 22°C and a humidity level of 60–70%. Animals had ad libitum access to food and water.

### Animal ethics statement

Experiments involving the care, bleeding and injection of mice with various venoms were reviewed and approved by the Institutional Animal Care and Use Committee of Chang Gung University (Permit Number: CGU14-024). The protocol of animal study on mice was based on the guidelines given by the Council for International Organizations of Medical Sciences (CIOMS)[19].

### Median lethal dose (LD_50_) assay

Groups of mice (n = 5/group) with a defined weight range (20–25 g) were subcutaneously injected with 0.1 ml of sterile saline solution containing different doses of venom. Six groups of mice were used to conduct this assay per venom. Only one dose was given to each mouse in this experiment. The dosage ranges of *N*. *kaouthia*, *N*. *siamensis* and *O*. *hannah* venom were 0.2–0.45, 0.4–0.7, 0.7–1.2 mg/kg, respectively. LD_50_ values were determined by recording deaths 24 hours after injection. The LD_50_ of each venom was calculated using Probit analysis [20] and showed the median with 95% confidence interval.

### Median effective dose (ED_50_) assay

This test involves incubation of a challenge dose, minimal lethal dose (MLD), of venom with different volumes of the antivenom, adjusted to a constant volume with saline solution. The mixtures were incubated for 30 minutes at 37°C, then 0.1-ml aliquots of each mixture were injected subcutaneously into groups of mice (n = 5/group) with a defined weight range (20–25 g). Mice in the control group were injected with a saline solution containing the challenge dose of venom alone, which induces 100% lethality. ED_50_ values were determined by recording deaths 24 hours after injection. The antivenom was considered ineffective when none of mice, administered with maximum amount of antivenom (0.1 ml), survived. The ED_50_ of each venom was calculated using Probit analysis [20] and presented as the median with 95% confidence interval. The neutralizing capacity expressed as ED_50_ and ER_50_ (median effective ratio), which are defined as the amount of antivenom that gives 50% survival of venom-challenged mice (for ED_50_) and the ratio of amount of venom to the volume dose of antivenom that keep 50% alive of mice (for ER_50_). Another term called “potency”, expressed as the amount of venom that is completely neutralized per milliliter of antivenom, was calculated as previously described [21, 22].

### Fractionation of venom proteins

Venom proteins of *N*. *kaouthia*, *N*. *siamensis*, *O*. *hannah* and *N*. *atra* were respectively separated by reverse-phase high-performance liquid chromatography (RP-HPLC) as previously described [23]. Briefly, crude venom (500 μg protein) was dissolved at 10 mg/mL in aqueous 0.1% trifluoroacetic acid (TFA) and 5% acetonitrile (ACN), and separated by RP-HPLC using a Suppelco Discovery 300 Å C18 (4.6 × 150 mm, 3 μm particle size) column. Flow rate was set to 1 mL/min, and the column was developed with a linear gradient of 0.1% TFA in water (solution A) and 0.1% TFA in ACN (solution B) as follows: isocratic 5% B for 5 minutes, followed by linear gradients of 5−40% B for 95 minutes, 40−70% B for 20 minutes, 70% B for 10 minutes, and re-equilibration with 5% B for 10 minutes. Peaks were detected by monitoring absorbance at 214 nm. Chromatographic fractions were collected manually, dried using a SpeedVac, and then stored at -20°C.

Each fraction was dissolved in sample buffer (125 mM Tris, 25% glycerol, 10% 2-mercaptoethanol, 4% SDS, 0.05% bromophenol blue), and one-half of each sample was analyzed by sodium dodecyl sulfate-polyacrylamide gel electrophoresis (SDS-PAGE) on 15% gels. The location of proteins in SDS-PAGE gels was visualized by Coomassie Brilliant Blue staining.

### In-gel tryptic digestion

After staining with Coomassie Brilliant Blue dye, the relatively abundant protein bands were excised from the gel and subjected to in-gel tryptic digestion, as described by Lin et al. [24]. Briefly, gel pieces were destained three times with 40% ACN containing 25 mM ammonium bicarbonate for 15 minutes each, reduced by incubating with 5 mM dithiothreitol at 60°C for 30 minutes, and then alkylated by incubating with 15 mM iodoacetamide at room temperature in the dark for 30 minutes. Proteins in the processed gel pieces were digested with freshly prepared trypsin solution containing 20 μg/mL of trypsin (Promega, Madison, WI, USA) in 25 mM ammonium bicarbonate at 37°C for 16 hours, then extracted with 100% ACN containing 1% TFA. Finally, the extracted tryptic peptides were concentrated by SpeedVac and stored at -20°C before use.

### LC-MS/MS analysis

Each peptide sample was reconstituted with 0.1%formic acid (FA), and then analyzed on a nano-LC–LTQ-Orbitrap Hybrid Mass Spectrometer (Thermo Fisher, San Jose, CA, USA), as described previously [25]. Briefly, the sample was loaded across a trap column (Zorbax 300SB-C18, 0.3 × 5 mm; Agilent Technologies, Wilmington, DE, USA) at a flow rate of 0.2 μL/min in HPLC buffer (0.1% FA), and separated on a resolving 10-cm analytical C18 column (inner diameter, 75 μm) using a 15-μm tip (New Objective, Woburn, MA, USA). The peptides were eluted using a linear gradient of 0–10% HPLC elution buffer (99.9% ACN containing 0.1% FA) for 3 minutes, 10–30% buffer B for 35 minutes, 30–35% buffer B for 4 minutes, 35–50% buffer B for 1 minute, 50–95% buffer B for 1 minute and 95% buffer B for 8 minute, with a flow rate of 0.25 μL/min across the analytical column. The resolution of the Orbitrap is 30,000, and the ion signal of (Si(CH_3_)_2_O)_6_H+ at 445.120025 (m/z) was used as a lock mass for internal calibration. A procedure that alternated between one MS scan followed by six MS/MS scans for the 10 most abundant precursor ions in the MS scan was applied. The m/z values selected for MS/MS were dynamically excluded for 180 seconds. For MS scans, the m/z value of the scan range was from 400 to 2000 Da. For MS/MS scans, more than 1 × 10^4^ ions were accumulated in the ion trap to generate MS/MS spectra. Both MS and MS/MS spectra were acquired using one scan with maximum fill-times of 1000 and 100 ms for MS and MS/MS analysis, respectively.

### Database searching and bioinformatics analysis

Raw MS data files were analyzed using Proteome Discoverer Software (version 1.3.0.339; Thermo Fisher, San Jose, CA, USA) and searched against an in-house–generated Squamata database originated from the UniProt database using the MASCOT search engine (version 2.2; Matrix Science, London, UK). The enzyme specificity parameter was set to “trypsin”, and one missed cleavage was allowed. Carbamidomethylation of cysteines was set as a static modification, and oxidations of methionine, acetyl (protein N-term) and Gln- > pyro-Glu (N-term Q) were set as dynamic modifications. The tolerance of MS was 10 ppm and that of MS/MS was 0.5 Da. The decoy database search approach was assessed for peptide identification, and the criteria of target false discover rate (FDR) was estimated to be <0.01 in this study. Each reported protein ID should have at least two peptide presenting in the sample, and at least one is the unique peptide for the reported protein.

### Western blot analysis

HPLC-fractionated samples (totally 200 μg) were resolved by SDS-PAGE, transferred onto polyvinylidene difluoride (PVDF) membranes, and then probed by incubating with 1:5000 (v/v) dilution of antivenom (stock solution, 80 mg/ml) at 4°C for 16 hours. Antivenom-reactive proteins were detected by incubating for 1 hour with alkaline phosphatase-conjugated anti-horse IgG secondary antibodies (Santa Cruz Biotechnology) and visualized using the CDP-Star Western Blot Chemiluminescence Reagent (PerkinElmer, Boston, MA, USA) with fluorescence detection.

## Results

### Pre-clinical evaluation of the cross-neutralization ability of FNAV

The lethality of the three Southeast Asian cobra venoms, measured as the subcutaneous (s.c.) LD_50_, was evaluated in a mouse model; the results are shown in **Table 1**. The LD_50_ of venom proteins from *N*. *kaouthia*, *N*. *siamensis*, and *O*. *hannah* were determined to be 0.34, 0.56, and 0.98 μg/g, respectively. Neutralization assays performed in mice injected with the minimum lethal dose of venom proteins from each cobra showed that FNAV effectively prevented death of mice induced by venoms of *N*. *kaouthia* and *N*. *siamensis*; ED_50_ values were 4.02 μL/mouse (Potency = 0.49 mg/ml) for *N*. *kaouthia* and 18.33 μL/mouse (Potency = 0.23 mg/ml) for *N*. *siamensis*. However, the lethality of *O*. *hannah* venom was not neutralized by FNAV, even at maximum dose of 100 μL/mouse (**Table 1**).

**Table 1:** Cross-neutralization ability of FNAV against venoms from *N*. *kaouthia*, *N*. *siamensis* and *O*. *hannah*.

Venom	Lethality		FNAV		
	LD_50_^a^ (μg/g)	MLD^b^ (μg/g)	ED_50_ (μL/mouse)	ER_50_ (mg/ml)	Potency^c^ (mg/ml)
*Naja kaouthia*	0.34 (0.22–0.39)	0.471 ± 0.02	4.02 (1.27–5.43)	2.19 (1.16–6.91)	0.49
*Naja siamensis*	0.56 (0.35–0.62)	0.756 ± 0.05	18.33 (0.59–1.04)	0.85 (0.65–1.43)	0.23
*Ophiophagus hannah*	0.98 (0.75–1.08)	1.325 ± 0.08	NE^d^	NE	NE

^a^ LD_50_: Median Lethal Dose—the dose of venom that induces lethality in 50% of subcutaneously injected mice (20–25 g). The values in parentheses are 95% confidence limits.

^b^ MLD: Minimum Lethal Dose—the lowest dose of venom that induces lethality in 100% of subcutaneously injected mice.

^c^ Potency: defined as the amount of venom in milligram was completely neutralized (100% protection) per milliliter of antivenom.

^d^ NE: Display ineffective.

### Proteomic analysis of venom proteomes of the four Southeast Asian cobras

**Fig 1:**
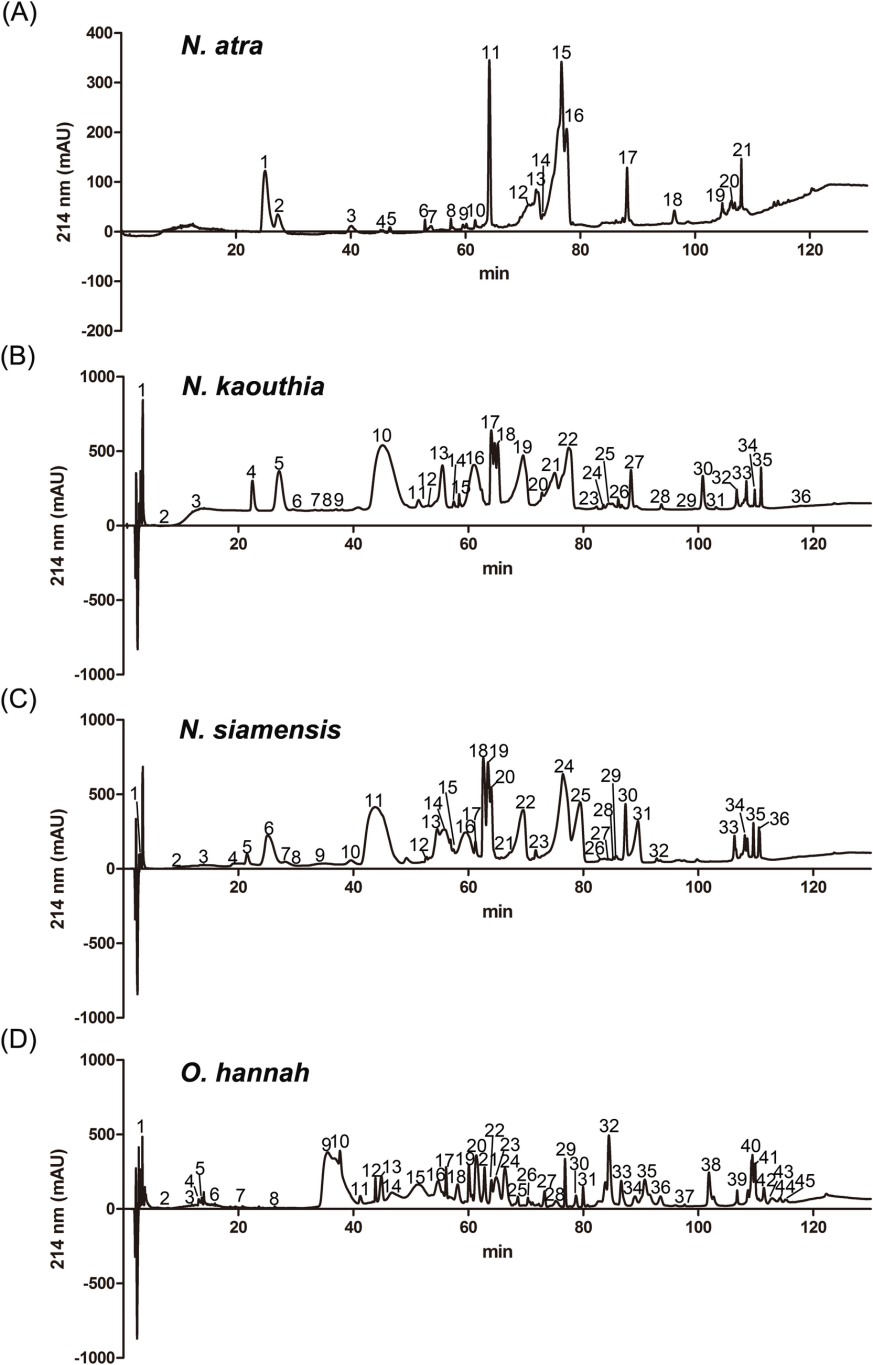
RP-HPLC separation of proteins from each cobra venom. The crude venoms of (A) *N*. *atra*, (B) *N*. *kaouthia*, (C) *N*. *siamensis*, and (D) *O*. *hannah* were fractionated on a C_18_ column; each fraction was collected manually. Shown are the chromatographic patterns for the four HPLC-separated venoms.

**Fig 2:**
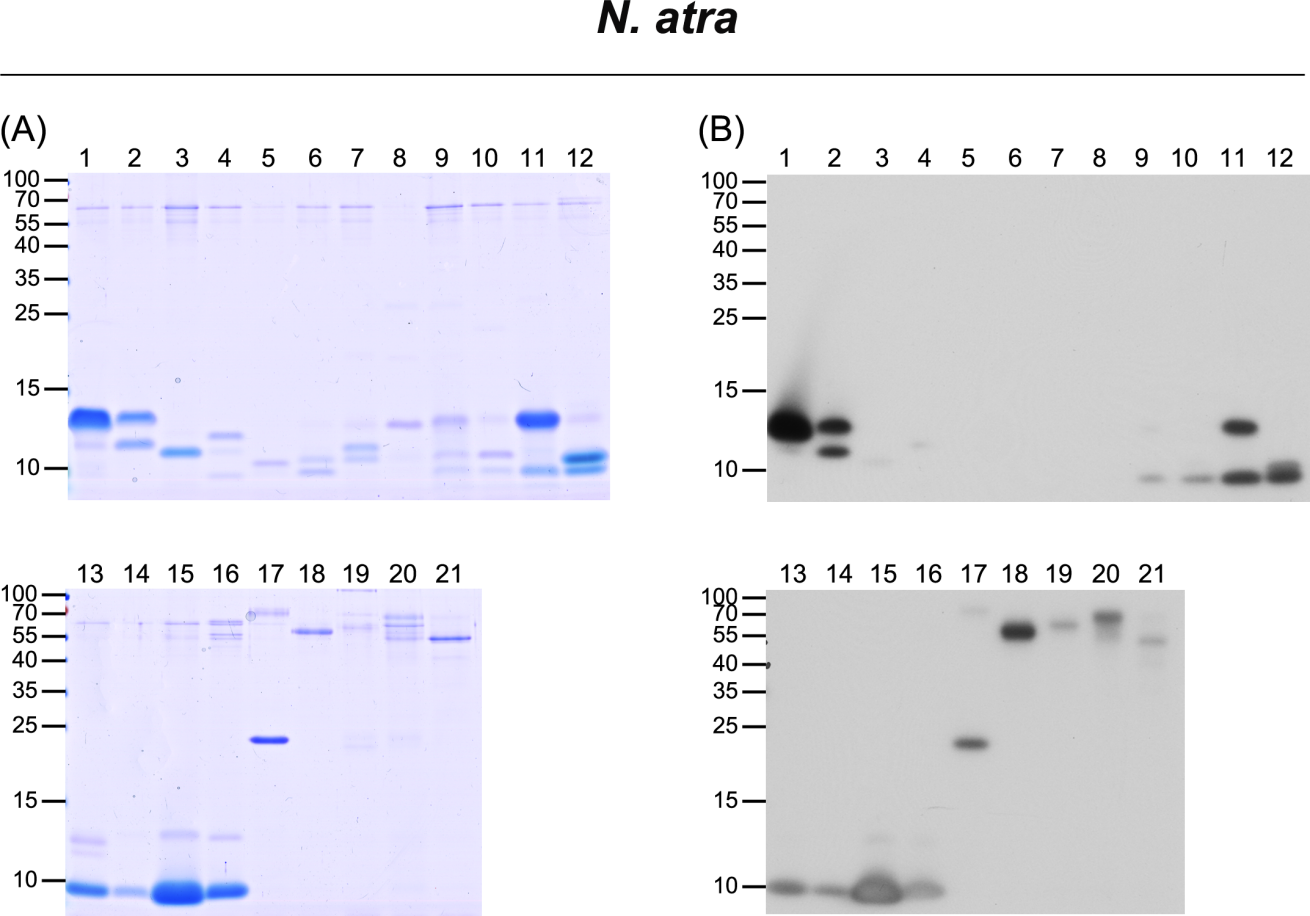
Characterization of HPLC-separated, FNAV-recognizable *N*. *atra* venom proteins by LC-MS/MS and Western blot analyses. HPLC-separated fractions of *N*. *atra* venom proteins were analyzed by SDS-PAGE, followed by (A) Coomassie Blue staining and (B) Western blotting using FNAV as a probe.

**Fig 3:**
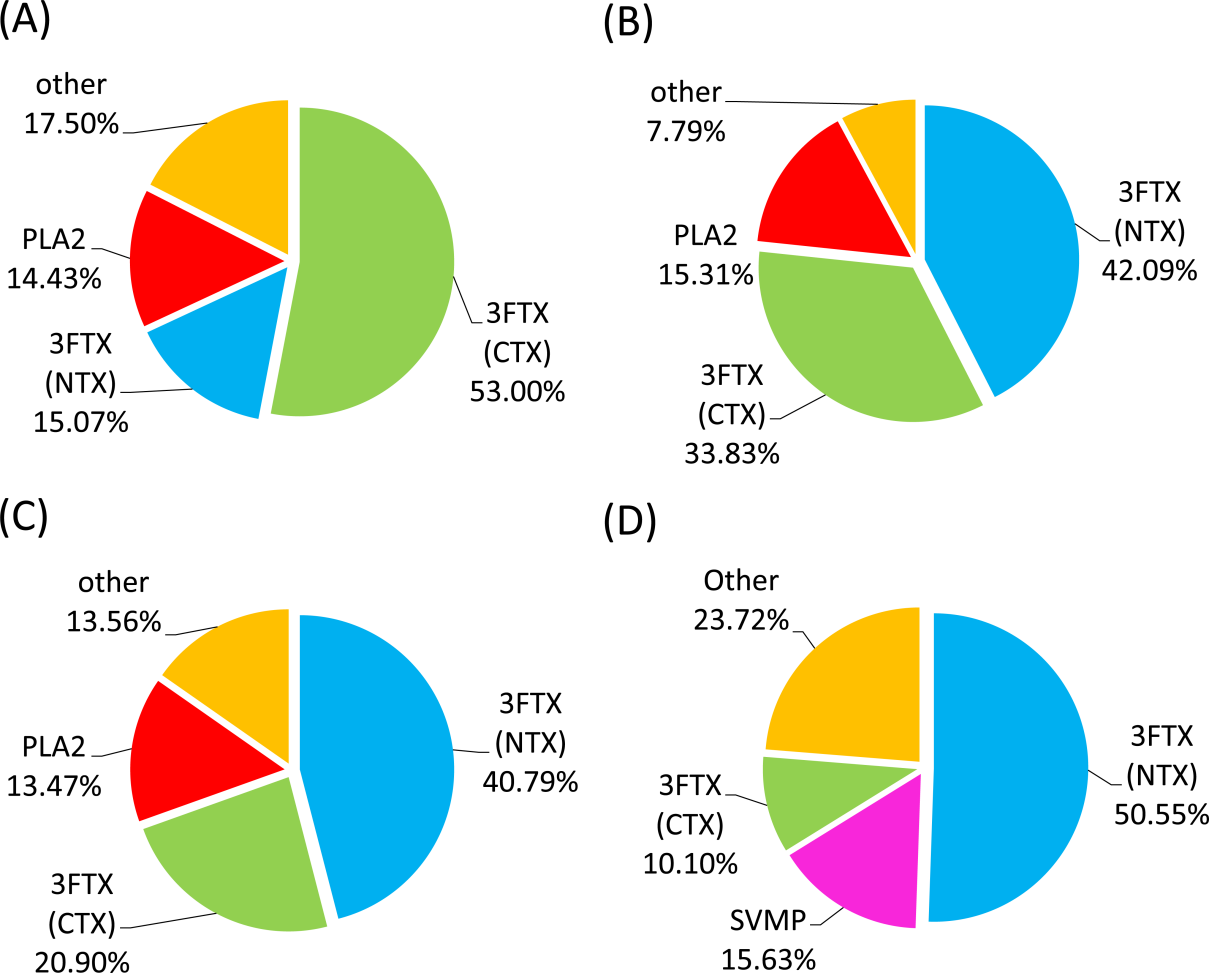
Pie charts showing the relative abundance of cobra venom protein families. The composition of venom proteins from (A) *N*. *naja*, (B) *N*. *kaouthia*, (C) *N*. *siamensis*, and (D) *O*. *hannah*. Results are based on venom protein identification and HPLC peak intensity, and show the major protein components, expressed as a percentage.

For the venom of *N*. *atra*, 21 fractions were collected from HPLC analysis (**Fig 1A**), and a total of 36 protein bands were assessed by LC-MS/MS for protein identification (**Fig 2A** and **S1 Fig**). Protein identification results are summarized in **Table 2**; additional detailed information is provided in **S1 Table**. The identified proteins belonged to eight protein families: three-finger toxin (3FTX), phospholipase A2 (PLA_2_), cysteine-rich secretory protein (CRISP), ohanin/vespryn (O/V), snake venom metalloproteinase (SVMP), venom nerve growth factor (VNGF), 5’ nucleotidase (5NT), and L-amino acid oxidase (LAAO). The 3FTX family proteins could be further categorized into the sub-families, cytotoxin (CTX), neurotoxin (NTX), and muscarinic toxin (MTX). Among these, CTX (53%), NTX (15%) and PLA_2_ (14%) were the dominant components identified in the *N*. *atra* venom proteome (**Fig 3A** and **S2 Table**).

**Table 2:** Summary of proteins identified in four venoms by LC-MS/MS analysis.

*N*. *atra* venom		*N*. *kaouthia* venom				*N*. *siamensis* venom				*O*. *hannah* venom			
Gel site	Protein Family	Gel site	Protein Family	Gel site	Protein Family	Gel site	Protein Family	Gel site	Protein Family	Gel site	Protein Family	Gel site	Protein Family
1–1	3FTX (NTX)	4–1	PLA_2_	18–5	3FTX (CTX)	4–1	LAAO	28–2	PLA_2_	9–1	3FTX (NTX)	38–1	SVMP
1–2	3FTX (NTX)	5–1	Unknown	19–1	Unknown	5–1	PLA_2_	29–1	CRISP	10–1	3FTX (NTX)	38–2	SVMP
2–1	PLA_2_	5–2	Unknown	19–2	Unknown	6–1	3FTX (NTX)	29–2	CRISP	11–1	3FTX (NTX)	40–1	SVMP
2–2	3FTX (NTX)	5–3	PLA_2_	19–3	Unknown	6–2	3FTX (NTX)	29–3	PLA_2_	12–1	3FTX (NTX)	40–2	SVMP
3–1	3FTX (NTX)	5–4	3FTX (NTX)	19–4	3FTX (CTX)	7–1	Unknown	29–4	3FTX (CTX)	13–1	3FTX (NTX)	41–1	SVMP
4–1	3FTX (MTX)	6–1	PLA_2_	20–1	Unknown	7–2	3FTX (NTX)	30–1	CRISP	14–1	3FTX (NTX)	42–1	SVMP
4–2	Unknown	8–1	Unknown	20–2	VNGF	9–1	3FTX (NTX)	30–2	3FTX (CTX)	15–1	SVMP	43–1	SVMP
5–1	3FTX (NTX)	8–2	Unknown	20–3	Unknown	10–1	3FTX (NTX)	31–1	CRISP	15–2	3FTX (NTX)	44–1	Unknown
6–1	3FTX (NTX)	8–3	3FTX (NTX)	20–4	3FTX (CTX)	11–1	3FTX (NTX)	31–2	3FTX (NTX)	16–1	3FTX (NTX)	45–1	Unknown
6–2	3FTX (NTX)	8–4	3FTX (NTX)	21–1	PLA_2_	12–1	3FTX (NTX)	32–1	Unknown	16–2	Kunitz	-	-
7–1	3FTX (NTX)	9–1	Unknown	21–2	3FTX (CTX)	13–1	3FTX (NTX)	33–1	PDE	17–1	Unknown		
7–2	3FTX (NTX)	9–2	3FTX (NTX)	21–3	3FTX (CTX)	14–1	3FTX (NTX)	33–2	5NT	17–2	Unknown		
8–1	VNGF	10–1	3FTX (NTX)	22–1	O/V	15–1	Unknown	34–1	5NT	17–3	3FTX (NTX)		
9–1	PLA_2_	11–1	3FTX (NTX)	22–2	3FTX (CTX)	15–2	VNGF	34–2	CVF	17–4	3FTX (NTX)		
9–2	PLA_2_	11–2	3FTX (NTX)	23–1	3FTX (CTX)	15–3	3FTX (NTX)	35–1	SVMP	18–1	3FTX (NTX)		
9–3	3FTX (CTX)	12–1	3FTX (NTX)	24–1	Unknown	16–1	PLA_2_	36–1	SVMP	18–2	3FTX (NTX)		
10–1	PLA_2_	12–2	3FTX (NTX)	24–2	CRISP	16–2	3FTX (NTX)	-	-	19–1	Unknown		
10–2	3FTX (MTX)	13–1	SVMP	24–3	O/V	17–1	PLA_2_			19–2	PLA_2_		
10–3	3FTX (CTX)	13–2	PLA_2_	24–4	3FTX (NTX)	17–2	3FTX (MTX)			19–3	3FTX (CTX)		
11–1	PLA2	13–3	3FTX (NTX)	25–1	Unknown	18–1	Unknown			20–1	3FTX (CTX)		
11–2	3FTX (CTX)	14–1	PLA_2_	25–2	CRISP	18–2	PLA_2_			21–1	PLA_2_		
12–1	3FTX (CTX)	14–2	3FTX (NTX)	25–3	PLA_2_	18–3	3FTX (CTX)			21–2	3FTX (CTX)		
12–2	3FTX (CTX)	14–3	3FTX (NTX)	25–4	3FTX (NTX)	19–1	PLA_2_			22–1	3FTX (CTX)		
13–1	VNGF	14–4	3FTX (MTX)	26–1	CRISP	19–2	3FTX (CTX)			23–1	3FTX (CTX)		
13–2	VNGF	15–1	VNGF	26–2	3FTX (NTX)	20–1	Unknown			24–1	3FTX (NTX)		
13–3	3FTX (CTX)	15–2	3FTX (NTX)	27–1	SVMP	20–2	PLA_2_			25–1	3FTX (NTX)		
14–1	3FTX (CTX)	16–1	Unknown	27–2	CRISP	20–3	3FTX (CTX)			26–1	Unknown		
15–1	O/V	16–2	Unknown	28–1	CRISP	21–1	3FTX (CTX)			27–1	PLA_2_		
15–2	3FTX (CTX)	16–3	PLA_2_	29–1	SVMP	22–1	PLA_2_			28–1	3FTX (NTX)		
16–1	O/V	16–4	PLA_2_	30–1	SVMP	22–2	3FTX (CTX)			29–1	3FTX (NTX)		
16–2	3FTX (CTX)	16–5	3FTX (NTX)	31–1	SVMP	23–1	VNGF			30–1	Unknown		
17–1	CRISP	16–6	3FTX (NTX)	32–1	PDE	23–2	3FTX (CTX)			31–1	O/V		
18–1	SVMP	17–1	Unknown	32–2	5NT	24–1	O/V			32–1	CRISP		
19–1	5NT	17–2	PLA_2_	32–3	SVMP	24–2	3FTX (CTX)			33–1	SVMP		
20–1	LAAO	17–3	3FTX (NTX)	32–4	GPX	25–1	3FTX (CTX)			34–1	Unknown		
21–1	SVMP	17–4	3FTX (NTX)	33–1	LAAO	26–1	Unknown			35–1	Unknown		
-	-	18–1	Unknown	33–2	CVF	26–2	3FTX (CTX)			35–2	SVMP		
		18–2	Unknown	33–3	GPX	27–1	CRISP			35–3	O/V		
		18–3	PLA_2_	34–1	SVMP	27–2	3FTX (CTX)			35–4	CRISP		
		18–4	Unknown	35–1	SVMP	28–1	CRISP			36–1	SVMP		

**Fig 4:**
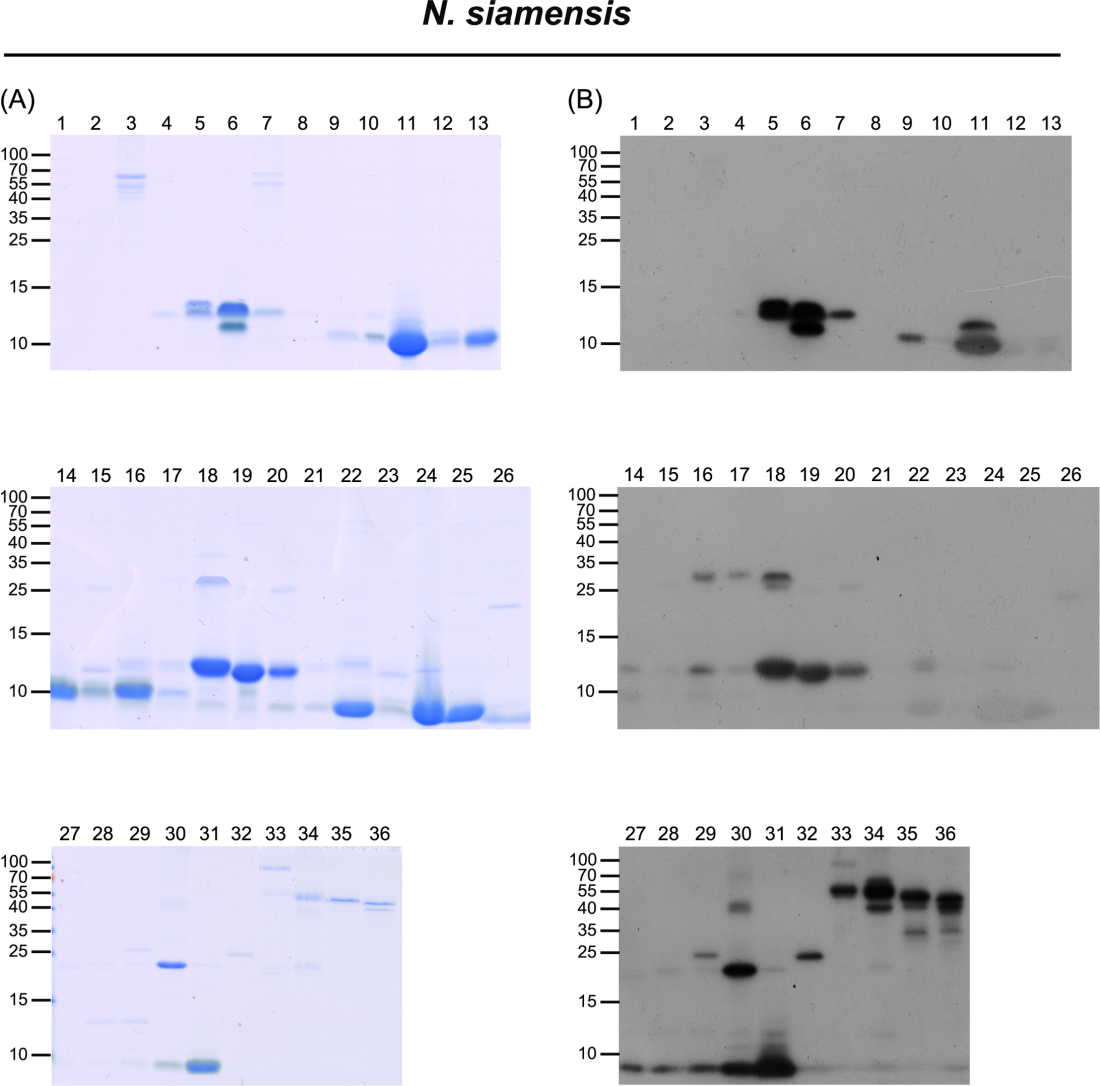
Characterization of HPLC-separated, FNAV-recognizable *N*. *kaouthia* venom proteins by LC-MS/MS and Western blot analyses. HPLC-separated fractions of *N*. *kaouthia* venom proteins were analyzed by SDS-PAGE, followed by (A) Coomassie Blue staining and (B) Western blotting using FNAV as a probe.

The venom of *N*. *kaouthia* yielded 36 protein fractions in HPLC analyses (**Fig 1B**). A total of 79 protein bands were analyzed by LC-MS/MS analysis for protein identification (**Fig 4A** and **S2 Fig**). Eleven different protein families were identified **(Table 2)**: 3FTX (NTX), 3FTX (CTX), PLA_2_, SVMP, CRISP, O/V, LAAO, VNGF, glutathione peroxidase (GPX), 3FTX (MTX), cobra venom factor (CVF), 5NT, and phosphodiesterase (PDE). Detailed information is shown in **S3 Table**. The top three major protein components were similar to those of the venom proteome of *N*. *atra*; however, the most abundant protein family was NTX (40%) rather than CTX (**Fig 3B** and **S2 Table**).

**Fig 5:**
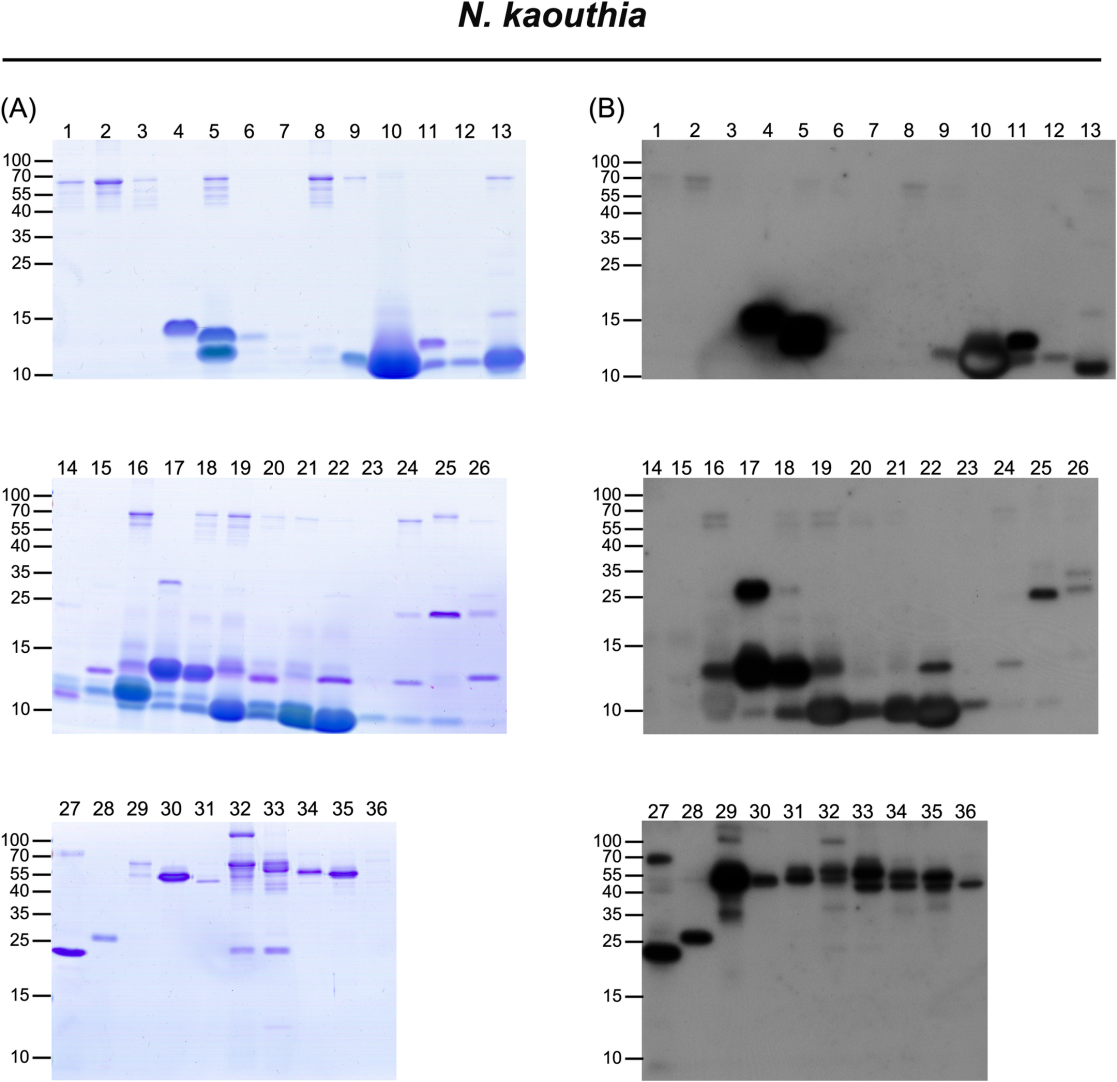
Characterization of HPLC-separated, FNAV-recognizable *N*. *siamensis* venom proteins by LC-MS/MS and Western blot analyses. HPLC-separated fractions of *N*. *siamensis* venom proteins were analyzed by SDS-PAGE, followed by (A) Coomassie blue staining and (B) Western blotting using FNAV as a probe.

Thirty-six fractions were obtained from HPLC separation of *N*. *siamensis* venom (**Fig 1C**), and 56 protein bands were selected for LC-MS/MS analysis (**Fig 5A** and **S3 Fig)**. The identified proteins could be categorized into nine protein families: 3FTX (NTX), 3FTX (CTX), PLA_2_, CRISP, SVMP, 5NT, 3FTX (MTX), VNGF, LAAO, O/V and CVF (**Table 2)**. Detailed information is shown in **S4 Table**. The relative abundances of protein components in this venom were very similar to those of *N*. *kaouthia*; the three major protein families were NTX (42%), CTX (33%) and PLA_2_ (15%), which collectively accounted for approximately 90% of *N*. *siamensis* venom proteins (**Fig 3C** and **S2 Table**).

**Fig 6:**
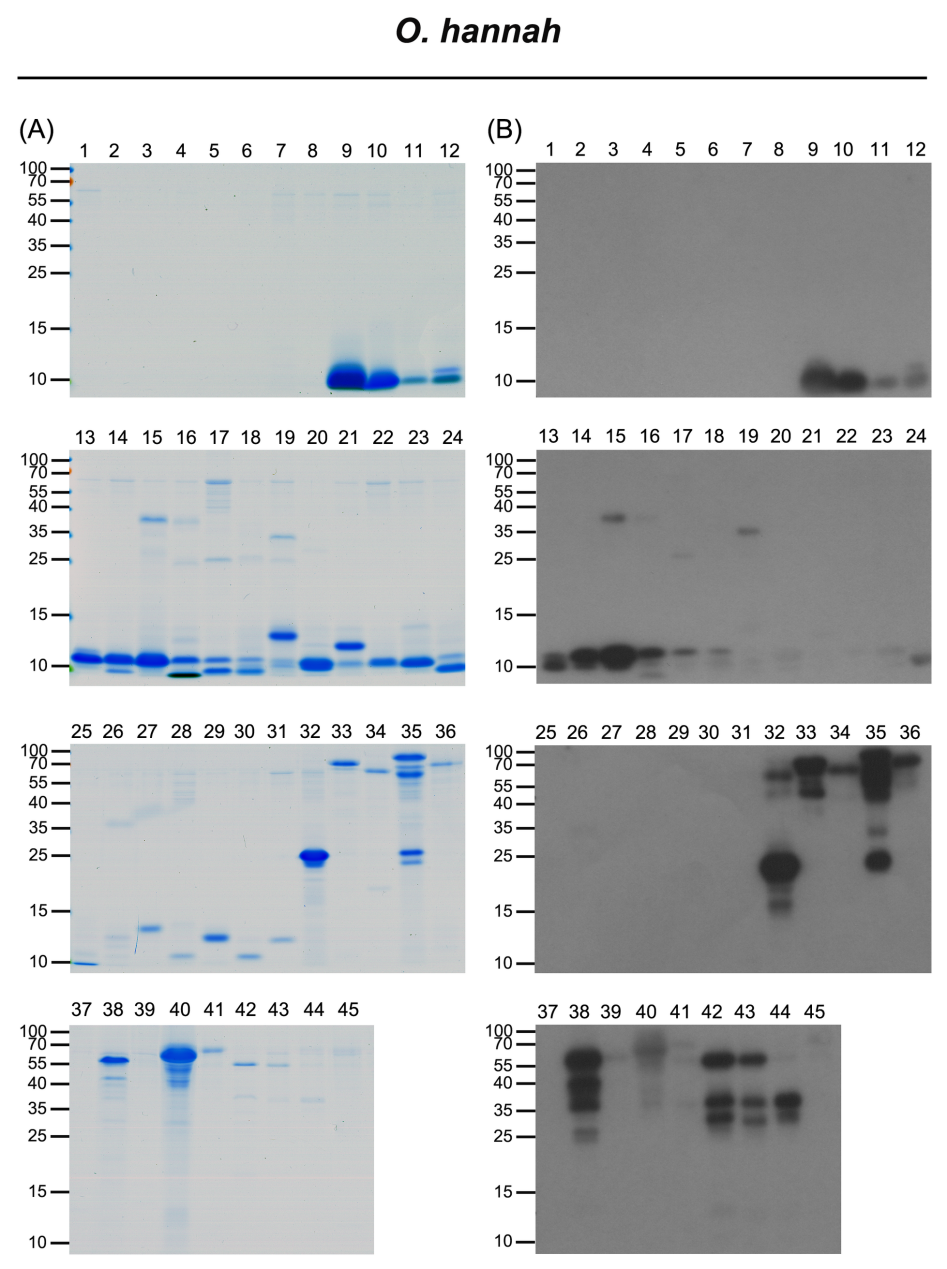
Characterization of HPLC-separated, FNAV-recognizable *O*. *hannah* venom proteins by LC-MS/MS and western blot analyses. HPLC-separated fractions of *O*. *hannah* venom proteins were analyzed by SDS-PAGE, followed by (A) Coomassie Blue staining and (B) Western blotting using FNAV as a probe.

The venom of *O*. *hannah* was initially separated into 45 fractions by HPLC analysis (**Fig 1D**), and further resolved into 49 protein bands for LC-MS/MS analysis (**Fig 6A** and **S4 Fig**). Only six protein families were identified (**Table 2**): 3FTX (NTX), SVMP, 3FTX (CTX), CRISP, PLA_2_, O/V and Kunitz-type protease inhibitor (Kunitz). The results of these analyses are shown in **S5 Table.** Unlike the case for the other three cobras, the three predominant protein components in *O*. *hannah* venom were NTX (50%), SVMP (15%) and CTX (10%), with PLA_2_ accounting for only 3–4% of the venom components (**Fig 3D** and **S2 Table**).

### Immunological profile of FNAV against venom proteins of the four cobras

Using Western blotting to investigate the immunoreactivity of FNAV towards the isolated protein fractions of the four snake venoms, we found that most of the components of *N*. *atra* venom were well recognized by FNAV (**Fig 2B**), including CTX (factions 11–16), NTX (fractions 1, 2), PLA_2_ (fraction 11), CRISP (fraction 17), and SVMP (fraction 18). Minor protein components present in fractions 3–8 did not, or weakly react with FNAV. The proteins identified in these fractions were categorized as belonging to NTX, MTX, and VNGF protein families (**Table 2**). Similarly, most of the major components of *N*. *kaouthia* and *N*. *siamensis* venoms were also reactive to FNAV. These included NTX (factions 10, 16), CTX (factions 19–22) and PLA_2_ (factions 4, 5, 17, 18) of *N*. *kaouthia* (**Fig 4B**), and NTX (factions 6, 11, 31), PLA_2_ (fractions 5, 18–20) and CTX (factions 27–30) of *N*. *siamensis* (**Fig 5B**). However, protein components in fractions 14 and 15 of *N*. *kaouthia* venom (**Fig 4B** and **Table 2**) and fractions 21, 22 (band 2), 23, 24, 25 and 26 (band 2) of *N*. *siamensis* venom (**Fig 5B** and **Table 2**) were weakly recognized by FNAV in Western blots, suggesting that these proteins may not be neutralized by FNAV, even though FNAV was capable of blocking the lethality of these two venoms in our animal model. Protein identification results (**Table 2**) showed that the major proteins in these fractions belonged to members of the CTX family. In addition to CTX, some NTX proteins detected in fractions 14–16 of *N*. *siamensis* venom also displayed very weak signals in Western blot analyses. For HPLC-fractionated *O*. *hannah* venom, NTX (fractions 9–18), CRISP (fraction 32, 35) and SVMP (fraction 33, 35, 38, 40, 42–44) immunoreacted strongly toward FNAV, whereas those in fractions 19 (band 3), 20, 21 (band 2), 22, 23, 28 and 29 were weakly detected, or not detected, by Western blotting (**Fig 6**and **Table 2**). The major protein in these fractions with lower immunereactivity was identified as β-cardiotoxin, a member of the CTX protein family (**S5 Table**). Moreover, the major proteins in fractions 19, 21 and 27, identified as PLA_2_ family proteins, also showed poor immunoreactivity to FNAV.

## Discussion

It should not come as a great surprise that species within the same genus would have evolved similar venom components. Thus, is conceivable that FNAV against the venom of *N*. *atra* could neutralize the snakebite of other *Naja* species. However, no dependable report has been provided to confirm this. In this study, we evaluated the therapeutic potential of FNAV against the venoms of three Southeast Asian venomous snakes and verified that FNAV neutralizes the lethal effects of *N*. *kaouthia* and *N*. *siamensis* venom in a mouse model.

Taiwan has more than four decades of experience in antivenom manufacture and refinement. Taiwan’s antivenoms are recognized for their quality and are known to be among the best antivenoms in the world. According to a previous clinical survey, most Taiwanese patients are successfully treated by administration of 1 vial of antivenom and are typically discharged without complications [18]. From the clinical perspectives, *N*. *atra* causes severe local necrosis but little flaccid paralysis in envenomed humans, however, the venom from cobra species (*N*. *simensis* and *N*. *kauothia*) used in this study predominately cause flaccid paralysis and severe local necrosis in envenomed subjects. Other relevant in-vivo tests such as minimum necrotic dose [26] and in-vitro nerve-muscle preparations [27, 28] should be performed before we can claim the clinical effectiveness of FNAV against *N*. *siamensis* and *N*. *kaouthia* snakebites [29]. For example, the neutralization of neurotoxicity by *N*. *kaouthia* monovalent antivenom (NKMAV) against three *N*. *kaouthia* from different regions has been evaluated by using the chick biventer cervicis nerve-muscle preparation system [30]. The alpha-neurotoxin-induced twitch depression could be prevented by pre-incubation of tissue with NKMAV, however, it didn’t fully restore the nerve-muscle contraction when the NKMAV added after the twitch depression onset. Therefore, this observation indicates that even though the FNAV has the ability to neutralize the lethality, or even the neurotoxicity in this neutralization assay, whether it can be used in real clinical setting (i.e. addition of antivenom after the symptoms onset) should be further confirmed. In addition, few studies have evaluated the immunoreactivity of antivenom against cobra venoms, and the results indicated the relatively lower neutralization ability toward NTX and CTX and PLA_2_ [31, 32].Further suggestions have been put forward to solve the low potency of cobra antivenom by preparing a purified venom-mixture containing only NTX, CTX and PLA_2_ as immunogens for antivenom preparation [31, 33]. Our current study on the immunoreactivity of FNAV also revealed that the NTX, CTX and PLA_2_ are the major immunological toxins in *N*. *kaouthia* and *N*. *siamensis* venom as well. The "cross-neutralization" phenomenon of FNAV against *N*. *kaouthia* and *N*. *siamensis* is potentially useful for further research into common antigenicity and perhaps the merging of a broader scale polyspecific antivenom, but more works need to be done to elucidate the immunological properties related to each major toxins involved in the pathophysiology and cross-neutralization.

Recently, three polyvalent antivenoms and one monovalent antivenom have been used to evaluate the neutralization potency against venoms of *N*. *kaouthia* and *N*. *siamensis* [7, 26]. The potency of Vin polyvalent antivenom (VPAV) and Bharat polyvalent antivenom, both raised against Indian *Naja naja*, *Bungarus caeruleus*, *Daboia russelli* and *Echis carinatus*, were determined to be 0.28 mg/ml and 0.37 mg/ml for *N*. *kaouthia* venom, respectively. To neutralize the venom of *N*. *siamensis*, the potency of the two antivenoms were reported to be 0.52 mg/ml and 0.14 mg/ml, respectively [26]. The neuro polyvalent antivenom (NPAV) was obtained from horse hyperimmunized with venoms of *N*. *kaouthia*, *O*. *hannah*, *Bungarus candidus* and *Bungarus fasciatus*. It has been demonstrated that both NPAV and the NKMAV have the same potency to neutralize the venoms of *N*. *Kaouthia* (0.94 mg/ml) and *N*. *siamensis* (1.15 mg/ml) [7]. Thus, these polyvalent antivenoms including FNAV reported here have the therapeutic potential for *N*. *kaouthia* and *N*. *siamensis* envenoming, and may serve as backup materials for snakebite treatment. Further analyses of these polyvalent antivenoms are needed to evaluate and compare their potential for clinical usage, such as the IgG content per antivenom, thermal stability, adverse effect and microbial contamination.

During our exploration of the FNAV-recognizable venom proteome of *N*. *atra*, we surprisingly found that protein components present in HPLC fractions 3–8 of *N*. *atra* venom (**Fig 2**) were weakly recognized by FNAV, considerably lower than others, despite the fact that FNAV was generated in horses hyperimmunized with *N*. *atra* venom. These proteins were identified by MS-based analysis as long neurotoxin homolog proteins (**S1 Table**). According to previous studies [34, 35], even though it has a characteristic of five disulfide bridges which can be classified as long neurotoxin, it exhibited the ability to inhibit acetylcholine-induced muscle contraction as cobrotoxin, a short neurotoxin; however, the degree of inhibition was less than half that of cobrotoxin. On the other hand, FNAV could react strongly toward fractions 1 and 2 from *N*. *atra* venom, which were identified as NTX and constituted ~15% of the whole venom. The short neurotoxin subtypes eluted in the early phase of RP-HPLC have been previously evaluated in mice models as the most lethal components in cobra venoms [13, 36]. These observations collectively suggest that the effectiveness of FNAV toward *N*. *atra* venom in mice models could be mainly contributed by the recognition of NTXs.

Our data showed that *N*. *kaouthia* and *N*. *siamensis* venoms could be cross-neutralized by FNAV. The classification of these species is still a matter of dispute, with some databases, such as *Uniprot* (http://www.uniprot.org/taxonomy/8649), considering *N*. *siamensis* to be the same as *N*. *kaouthia*. The data from our present study indicate the high similarity between the *N*. *kaouthia* and *N*. *siamensis* venoms; the LD_50_ of *N*. *kaouthia* and *N*. *siamensis* venoms differ only marginally (0.34 μg/g v.s. 0.56 μg/g, with overlapped 95% C.I. 0.22–0.39 v.s. 0.35–0.62, see **Table 1**) and their composition patterns in term of the major components (~40% neurotoxins, 15% PLA2 in particular, see **Fig 3**) are almost comparable (based on chromatogram and proteomes, see **Figs 1, 4 and 5**). In spite of these similarities, we also observed differences between their venom proteome. For example, the protein “hemorrhagic metalloproteinase-disintegrin-like kaouthiagin (P82942)” identified in fraction 30 from *N*. *kaouthia* venom was not detected *N*. *siamensis* venom. Additionally, *N*. *siamensis* venom contained a fewer amount of cytotoxin proteins as compared to *N*. *kaouthia* venom. These venom antigen variations probably led to the difference in the neutralization of FNAV tested in mice.

*N*. *kaouthia* is primarily distributed to Malaysia, Thailand and Vietnam, and its venom proteomes and toxicity in these countries have been previously reported [7, 37]. The lethality of *N*. *kaouthia* venom in these different regions is reportedly different, with that from Thailand being more venomous than Malaysian or Vietnamese venom. Furthermore, the Thai *N*. *kaouthia* venom contains higher amounts of long neurotoxins, while the Malaysian and Vietnamese specimens are particularly rich in cytotoxins. This geographical proteomic variation supported the observation that *N*. *kaouthia* venom from Thailand has higher neuromuscular depressant activity than that from Malaysia or Vietnam [30]. In the present study, we only tested the ability of FNAV to neutralize the venom of *N*. *kaouthia* from Thailand. Thus, if there are future hopes of using FNAV to treat *N*. *kaouthia* envenomation in Malaysia and Vietnam, the ability of FNAV to cross-neutralize *N*. *kaouthia* venom from Malaysia and Vietnam should be re-evaluated, notwithstanding the fact that these are the same species as *N*. *kaouthia* from Thailand. In addition, the neutralization of the foundation toxicity from these venoms by FNAV should be tested in the future as well.

*O*. *hannah* venom represents the only venom that could not be neutralized by FNAV in current study, although FNAV did cross-reacted intensely with the major lethal toxins (neurotoxins, which formed 50% of proteome based on our venom proteome and immunoprofiling analyses as shown in **Figs 3 and 6**and **S5 Table**). It was found that β-cardiotoxin, identified in the HPLC fractions 20–23, is one of venom proteins weakly recognized or even non-recognized by FNAV. Previous studies have reported that β-cardiotoxin is a natural exogenous β-blocker [38] that can bind to β1- and β2-adrenergic receptors, causing a dose-dependent decrease in heart rate. Intraperitoneal injection of this protein into mice induces labored breathing, impaired locomotion, and death within 30 minutes; however, the lethal dose of β-cardiotoxin is higher than 10 μg/g, suggesting that β-cardiotoxin might not be the major toxins of *O*. *hannah* venom. In addition, PLA_2_ in *O*. *hannah* venom also showed poor immunoreactivity to FNAV. It is well known that there are numerous PLA_2_ isoforms with different physiological/pathological functions in the snake venoms. Although these PLA_2_s show very high similarity in their three dimensional folding, their primary structures (amino acid sequences) can be varied significantly [39]. Theoretically, these sequence variations may confer distinct immunological properties for different PLA_2_s. We aligned and compared the sequences between PLA_2_s identified from different snakes, *O*. *hannah*, *N*. *atra*, *N*. *kaouthia* and *B*. *multicinctus*(**S5 Fig**). This analysis revealed that the sequence similarity is quite low between *O*. *Hannah* PLA_2_ and *N*. *atra* PLA_2_ (64%) or between *O*. *Hannah* PLA_2_ and *B*. *multicinctus* PLA_2_ (59%). However, the sequence similarity between *N*. *atra* PLA_2_ and *N*. *kaouthia* PLA_2_ is much higher (up to 95%). Therefore, this alignment analysis together with our immunological profiling data suggest that *O*. *Hannah* PLA_2_ might have distinct antigenic site(s) as compared with PLA_2_ from venoms of *N*. *atra*, *N*. *kaouthia* and *B*. *multicinctus*. Although we observed that the immunorecognition of FNAV toward β-cardiotoxin and PLA_2_ is weakly, the pharmacological activities of both β-cardiotoxin and PLA_2_ seem not to correlate with the major symptom (neurotoxicity) caused by *O*. *hannah* venom. Hence, the conflicting finding that FNAV could react strongly to the major neurotoxins yet failed to neutralize the lethality at the challenge dose used remains unresolved here and warrants further study. Furthermore, *O*. *hannah* is the only venomous snake whose whole genome has been sequenced [40]. Its venom transcriptome and proteome have been studied as well [23, 40–43]. These studies reported the presence of different amounts of LAAO family proteins in the *O*. *hannah* venom, which may be due to geographical variation of the venom. However, there were not any LAAO proteins identified in our *O*. *hannah* venom proteome. The exact reason(s) for this discrepancy is (are) currently unknown. One of the possible reasons is that the amount of LAAO protein in our *O*. *hannah* venom might be too low to be detected after the whole process for venom sample preparation and fractionation. Another possibility is that LAAO protein might have been degraded in the venom after long-term storage and thus could not be detected.

We have characterized the venom proteomes and FNAV-recognizable venom proteins of these four Southeast Asian snakes, *N*. *atra*, *N*. *kaouthia*, *N*. *siamensis* and *O*. *hannah*, allowing us to identify the major venom components, both FNAV-reactive and -unreactive. This information should advance our understanding of venom immunogenicity and facilitate further improvement of antivenom design, which allow us to predict the cross neutralization to the level of cobra specific toxins [12]. For venoms from three *Naja* species—*N*. *atra*, *N*. *kaouthia* and *N*. *siamensis*—the three major venom components were identified as CTX, NTX and PLA_2_, which also represent the dominant targets recognized by FNAV. The sequences of these three components are highly similar between each *Naja* species, and the major functions of them are responsible for the toxic effects, necrosis, and neurotoxicity observed in cobra-envenomed patients [44–47]. Our study further strengthens the previous report that CTX, NTX and PLA_2_ are the most abundant and medically-relevant toxin components in the venom of cobra species [13, 14, 31]. To extend the use of FNAV for treating life-threatening snake envenomations in areas with antivenom shortages, it would be ideal to determine the ability of FNAV to neutralize the venom from all *Naja* species. However, because the venoms from several countries are unavailable, we were only able to obtain venoms from three Naja species for the present study. Three other *Naja* species—*Naja naja*, *Naja nivea* and *Naja haje*—are important targets for further studies to evaluate the FNAV cross-neutralization ability. *N*. *naja* is mainly distributed in India, where a large proportion of global snakebites occur [4]. Snakebite mortality remains high in modern India, with approximately 40,000 deaths per year [48, 49]. On the other hand, *N*. *nivea* and *N*. *haje* live in Africa, where few antivenoms are available and antivenom is in short supply [50]. These two areas may urgently need new antivenoms to solve their local snakebite crises.

## Supporting information

S1 Fig Labeling of HPLC/SDS-PAGE-separated protein bands from *N*. *atra* venom for MS-based protein identification. (TIF)

S2 Fig Labeling of HPLC/SDS-PAGE-separated protein bands from *N*. *kaouthia* venom for MS-based protein identification. (TIF)

S3 Fig Labeling of HPLC/SDS-PAGE–separated protein bands from *N*. *siamensis* venom for MS-based protein identification. (TIF)

S4 Fig Labeling of HPLC/SDS-PAGE–separated protein bands from *O*. *hannah* venom for MS-based protein identification. (TIF)

S5 Fig The alignment analysis of amino acid sequence between phospholipase A2 from different venomous snakes. (A) *N*. *atra* versus *O*. *hannah*, (B) *B*. *multicinctus* versus *O*. *hannah*, and (C) *N*. *atra* versus *N*. *kaouthia*.
(TIF)

S1 Table Summary of protein identification results of *N*. *atra* venom by LC-MS/MS analysis. (XLSX)

S2 Table The relative abundance of proteins of different protein families showed as the percentage of the whole venom content. (XLSX)

S3 Table Summary of protein identification results of *N*. *kaouthia* venom by LC-MS/MS analysis. (XLSX)

S4 Table Summary of protein identification results of *N*. *siamensis* venom by LC-MS/MS analysis. (XLSX)

S5 Table Summary of protein identification results of *O*. *hannah* venom by LC-MS/MS analysis. (XLSX)
