# Supplementary figures and images for: Analysis of the efficacy of Taiwanese freeze-dried neurotoxic antivenom against Naja kaouthia, Naja siamensis and Ophiophagus hannah through proteomics and animal model approaches
Source: PLoS Negl Trop Dis. 2017 Dec 15;11(12):e0006138. doi: 10.1371/journal.pntd.0006138 (PMC5747474; doi:10.1371/journal.pntd.0006138)

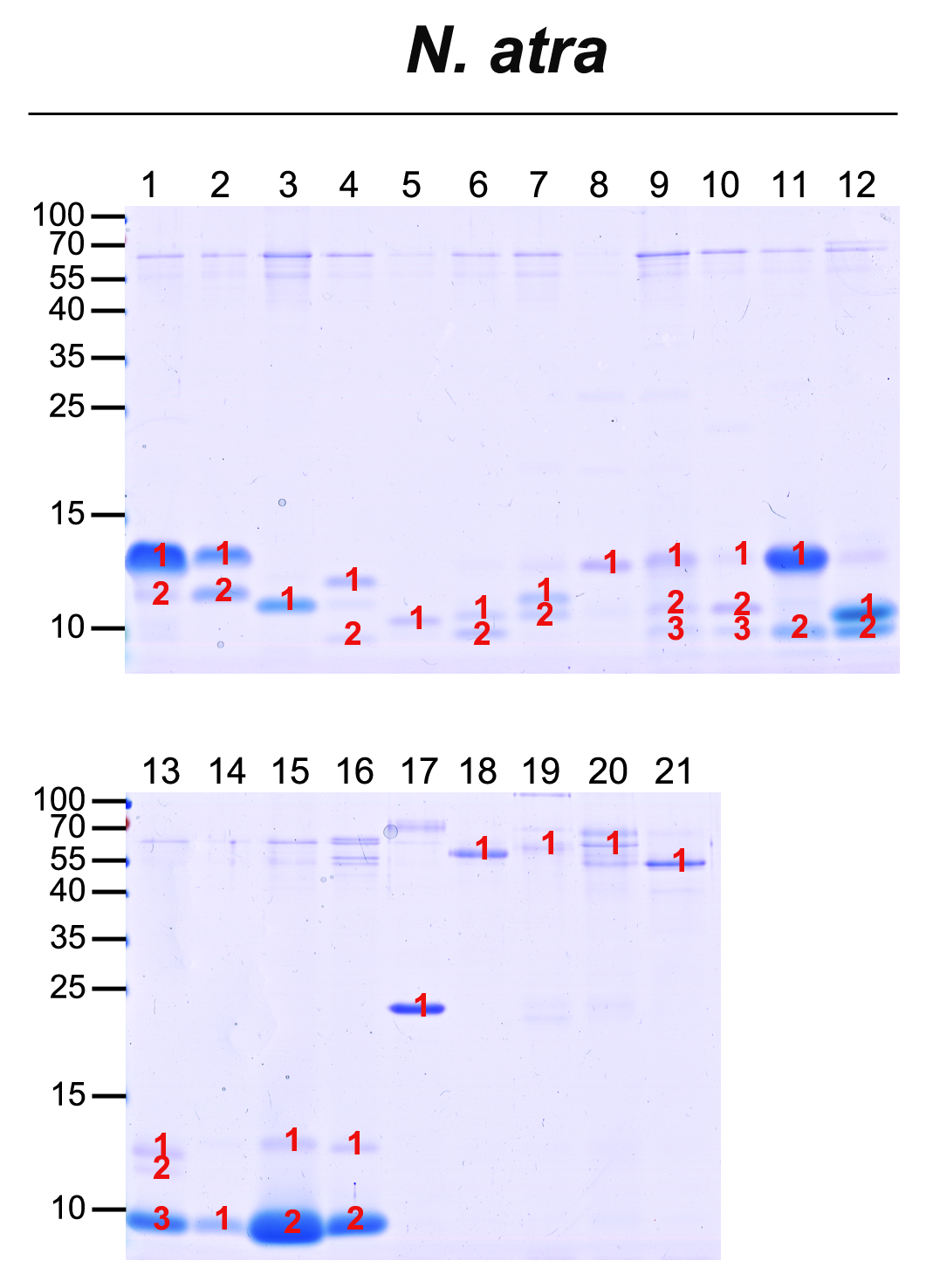

Supplement: S1 Fig — (TIF) [file pntd.0006138.s001.tif]

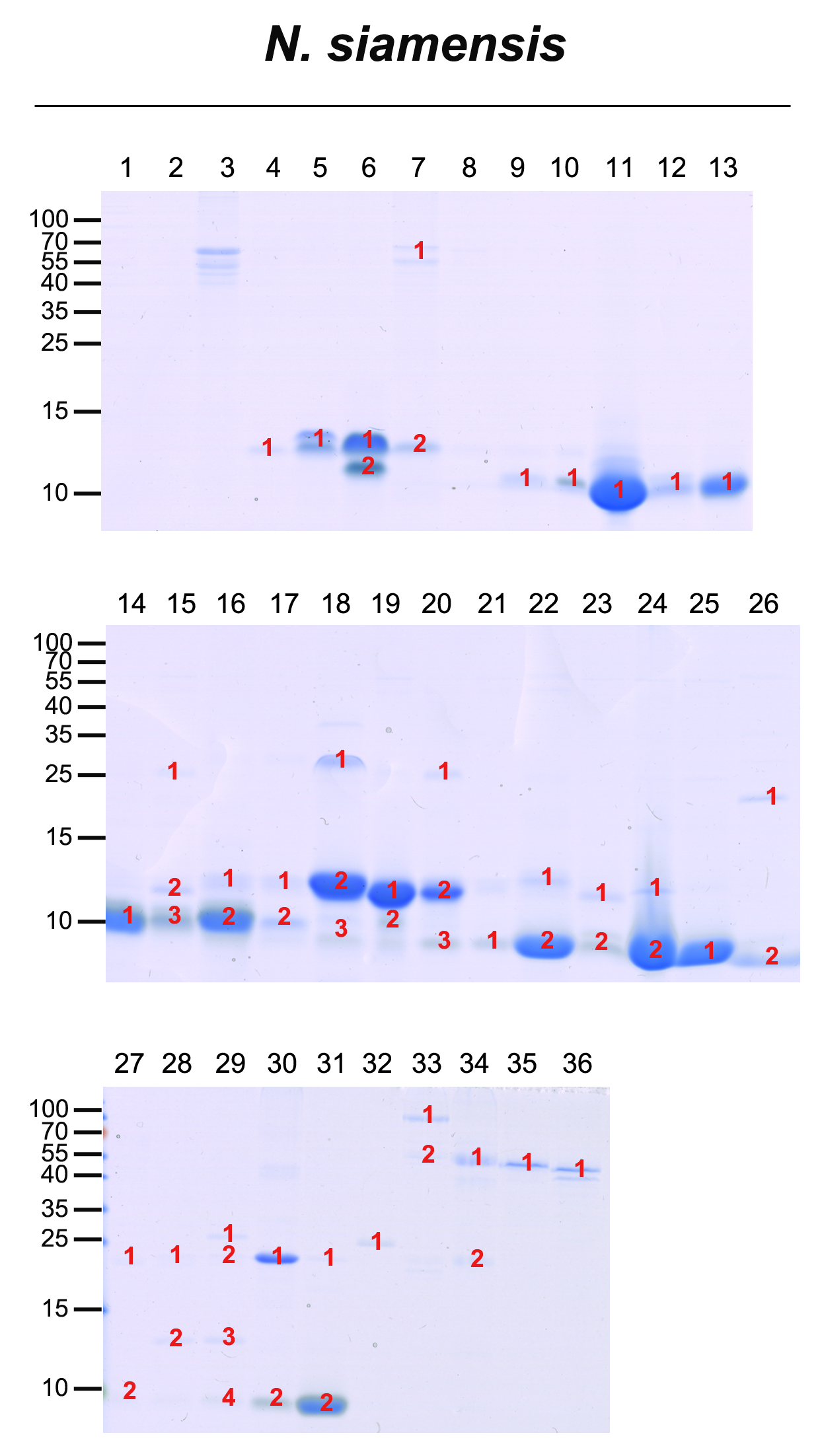

Supplement: S2 Fig — (TIF) [file pntd.0006138.s002.tif]

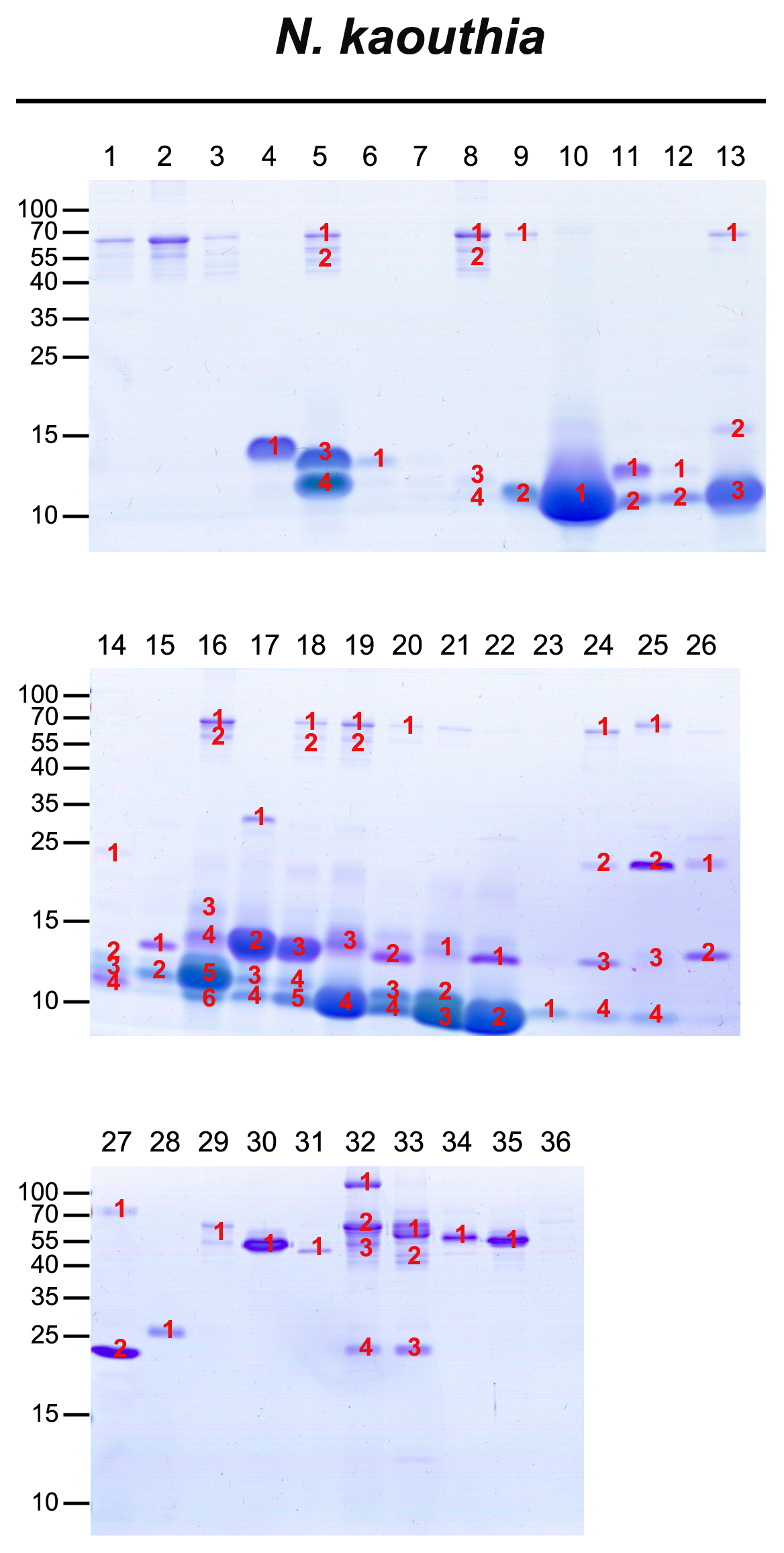

Supplement: S3 Fig — (TIF) [file pntd.0006138.s003.tif]

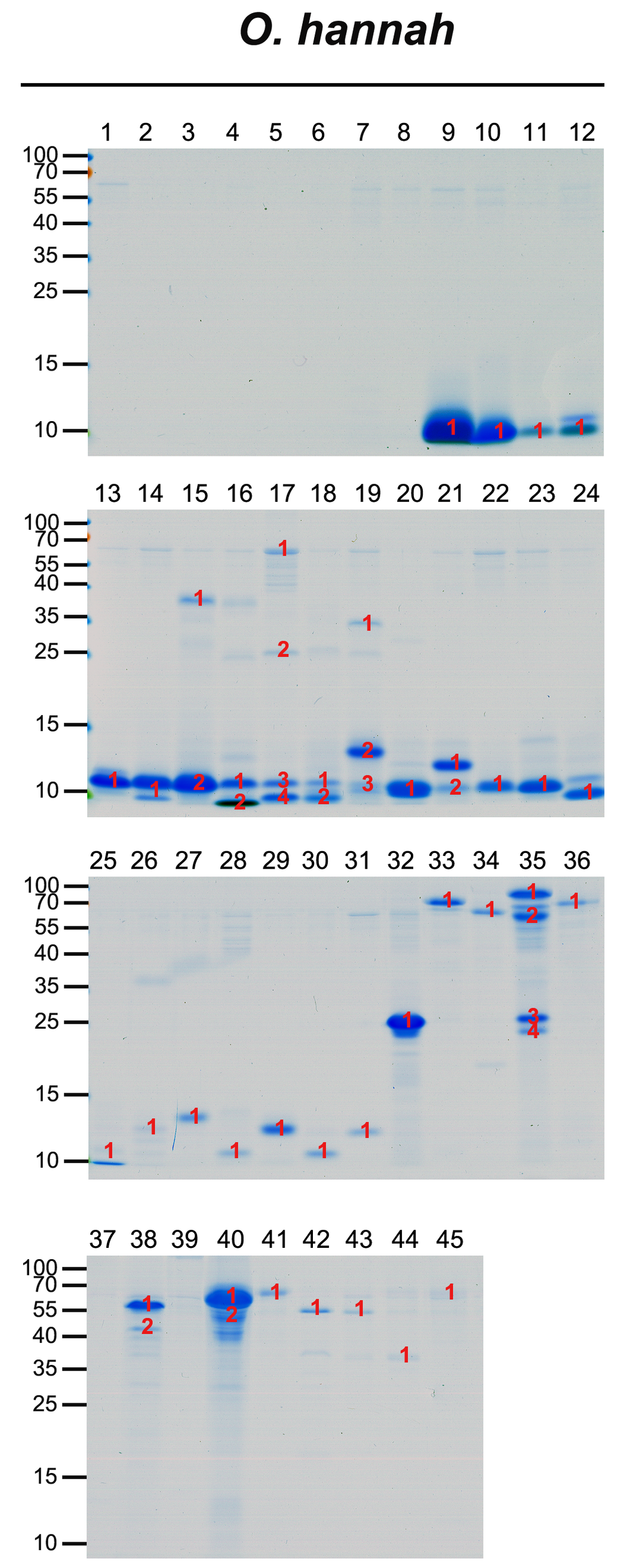

Supplement: S4 Fig — (TIF) [file pntd.0006138.s004.tif]

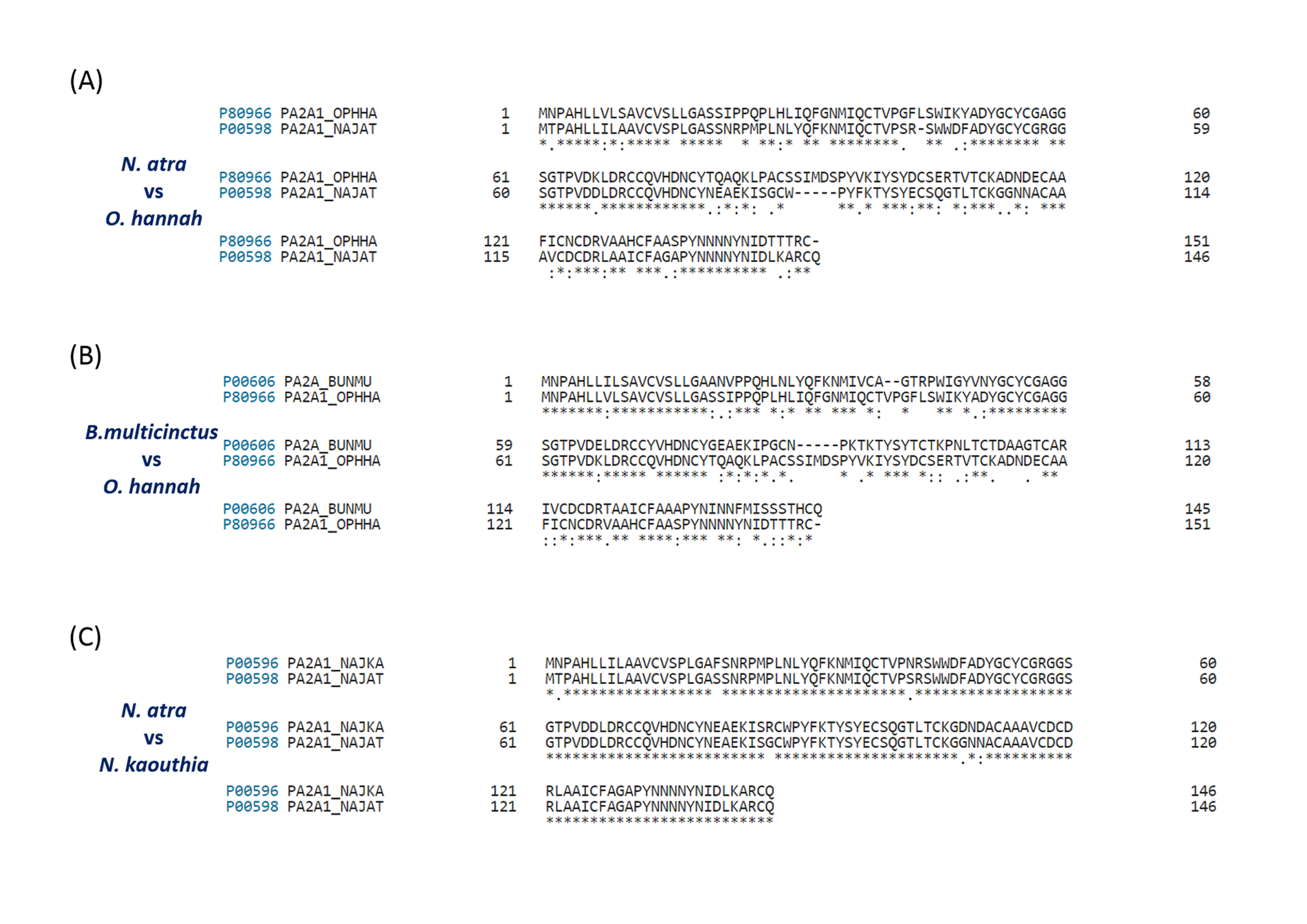

Supplement: S5 Fig — (A) N. atra versus O. hannah, (B) B. multicinctus versus O. hannah, and (C) N. atra versus N. kaouthia. (TIF) [file pntd.0006138.s005.tif]
